# Effect of Hydrogen Peroxide on the Antibacterial Substantivity of Chlorhexidine

**DOI:** 10.1155/2010/946384

**Published:** 2011-01-19

**Authors:** Shahriar Shahriari, Zahed Mohammadi, Mohammadi Mehdi Mokhtari, Rasoul Yousefi

**Affiliations:** ^1^Department of Endodontics, Hamedan University of Medical Sciences, Hamedan, Iran; ^2^Iranian Center for Endodontic Research (ICER), Tehran, Iran; ^3^Private Endodontic Practice, Tehran, Iran; ^4^Department of Bacteriology, Hamedan University of Medical Sciences, Hamedan, Iran

## Abstract

The purpose of this *in vitro* study was to assess the effect of hydrogen peroxide on the antibacterial substantivity of chlorhexidine (CHX). Seventy-five dentine tubes prepared from human maxillary central and lateral incisor teeth were used. After contamination with *Enterococcus faecalis* for 14 days, the specimens were divided into five groups as follows: CHX, H_2_O_2_, CHX + H_2_O_2_, infected dentine tubes (positive control), and sterile dentine tubes (negative control). Dentine chips were collected with round burs into tryptic soy broth, and after culturing, the number of colony-forming units (CFU) was counted. The number of CFU was minimum in the first cultures in all experimental groups, and the results obtained were significantly different from each other at any time period (*P* < .05). At the first culture, the number of CFU in the CHX + H_2_O_2_ group was lower than other two groups. At the other experimental periods, the CHX group showed the most effective antibacterial action (*P* < .05). Hydrogen peroxide group showed the worst result at all periods. In each group, the number of CFU increased significantly by time lapse (*P* < .05). In conclusion, H_2_O_2_ had no additive effect on the residual antibacterial activity of CHX.

## 1. Introduction

The major contribution of microorganisms in the induction and continuing of pulpal and periapical diseases has clearly been demonstrated in animal models and human studies [1–3]. Methods to reduce root canal microorganisms include thorough instrumentation, the use of an effective irrigating solution, and intracanal medicaments. Mechanical instrumentation alone does not result in a bacteria-free root canal system, and when the complex anatomy of the root canal system [4] is considered, this is not surprising. Furthermore, *ex vivo* and clinical evidence has revealed that mechanical instrumentation leaves significant portions of the root canal walls untouched [5], and complete elimination of bacteria by instrumentation alone is unlikely to occur [6]. Therefore, in order to remove residual tissue and to kill microorganisms, some form of irrigation and disinfection is needed. In cases with necrotic pulps as well as in retreatment cases, treatment should be performed in two visits, which is more time consuming than one-visit treatment [7]. In addition, it has been demonstrated that calcium hydroxide is ineffective against *Enterococcus faecalis* [8]. To overcome the aforementioned problems, an alternative protocol is to use antimicrobial agents that exhibit substantivity, that is, agents that can have a therapeutic effect for a prolonged period.

 Sodium hypochlorite (NaOCl) is the most commonly used root canal irrigation solution. Despite its excellent tissue-dissolving and antimicrobial abilities [9, 10], NaOCl possesses some drawbacks. One of its major drawbacks is its high surface tension, which limits its penetration into canal irregularities and the depth of dentinal tubules [11].

 CHX is a cationic biguanide that seems to act by Being adsorbed onto the cell wall of the microorganism and causing leakage of intracellular components. At low concentrations, small molecular weight substances will leak out, resulting in a bacteriostatic effect. At higher concentrations, CHX has a bactericidal effect due to precipitation and/or coagulation of the cytoplasm [12].

CHX has a unique feature in that dentine medicated with it acquires antimicrobial substantivity. The positively charged ions released by CHX can be adsorbed into dentine and prevent microbial colonization on the dentine surface for some time beyond the actual period of time of application of the medicament [13].

Hydrogen peroxide (H_2_O_2_) is another irrigation solution. It is an active agent that affects a wide range of organisms such as bacteria, yeast, fungi, viruses, and spores [14]. The antibacterial effect of HP involves hydroxyl radicals. The hydroxyl radical, being a potent oxidant, can react easily with macromolecules such as membrane lipids and DNA thus resulting in bacterial death [14]. 

Antibacterial substantivity of CHX has been demonstrated in many studies [13, 15–17]. Furthermore, two studies have revealed the synergistic antibacterial activity between CHX and H_2_O_2_ [18, 19]. However, the effect of H_2_O_2_ on the antibacterial substantivity of CHX has not been studied yet. Therefore, we decided to evaluate the effect of combining with H_2_O_2_ on the antibacterial substantivity of CHX.

## 2. Materials and Methods

**Figure 1:**
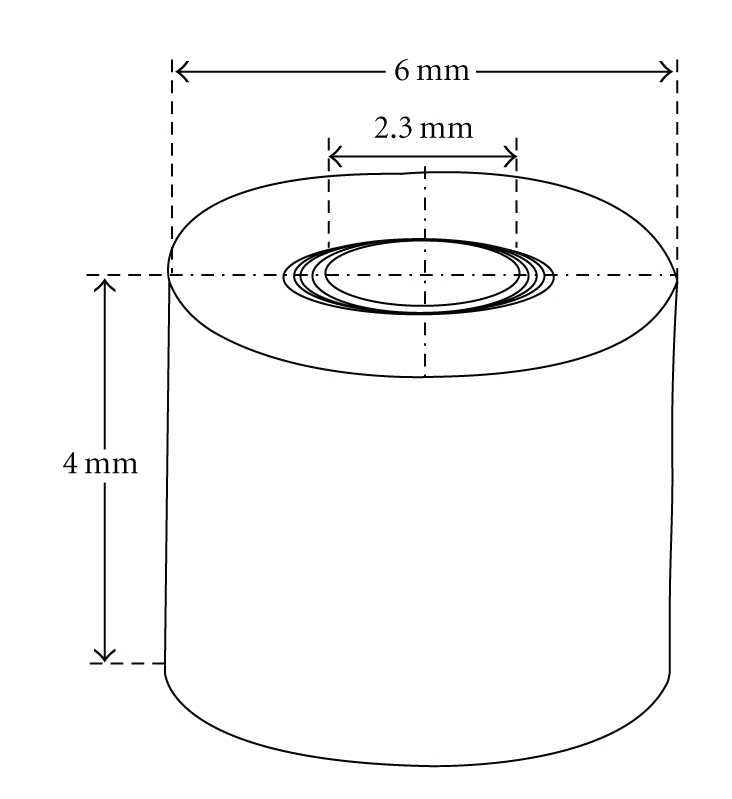
Schematic view of used dentin tubes (adopted from Mohammadi and Shahriari [15]).

The method used in the present study is a modification of the procedure previously described by Haapasalo and Orstavik [8]. Intact human central and lateral incisor teeth were used for this study. The teeth were kept in 0.5% NaOCl solution for up to 7 days. The clinical crown and apical third were removed from each tooth with a rotary diamond saw at 1000 rpm (Isomet Plus precision saw, Buehler, IL, USA) under water cooling. Cementum was removed by using polish paper (Ecomet 3, variable-speed grinder-polisher, Buehler, IL, USA), which resulted in a centre-holed piece of root dentin with a 6-mm outer diameter (Figure 1). The remained piece of each tooth was then cut into 4-mm thick slices with a diamond saw as above. The canals of the 4-mm blocks were enlarged (standardized) with an ISO 023 slow speed round bur. In order to prevent dehydration, all teeth and dentin slices were preserved in vials containing tap water during the procedures. Each dentin block (*n* = 75) was individually treated with 5.25% NaOCl and 17% EDTA (with pH 7.2) to remove the smear layer. The specimens were then placed in BHI broth (Oxoid, Basingstoke, UK) and autoclaved. To monitor the efficacy of the sterilization, they were then kept in an incubator at 37°C for 24 h. A total of 75 specimens were randomly divided into five groups as follows: Group 1 (15 specimens): 2% CHX; Group 2 (15 specimens): 3% H2O2; Group 3 (15 specimens): 2% CHX + 3% H2O2; Group 4 (15 specimens): positive control (infected dentin tubes); Group 5 (15 specimens): negative control (sterile dentin tubes). Isolated 24-h colonies of pure cultures of E. faecalis (ATCC 29212) were suspended in 5 mL of BHI. The bottles containing each specimen in Groups 1, 2, 3, and 4 were opened under laminar flow. Two milliliters of sterile BHI was removed with sterile pipettes and replaced with 2 mL of bacterial inoculum. The bottles were closed and kept at 37°C for 14 days, with the replacement of 1 mL of contaminated BHI for 1 mL of freshly prepared BHI every 2 days, to avoid medium saturation. After the contamination period, each specimen was removed from its bottle under aseptic conditions, and the canal was irrigated with 5 mL of sterile saline and dried with sterile paper points. In order to prevent contact of the medicament with the external surface, the outer surface of the specimens was covered with two layers of nail varnish. Thereafter, using decontaminated sticky wax, specimens were fixed at the bottom of wells of 24-well cell culture plates which also obliterated the apical surface of the root canal. Finally, the irrigating solutions were inserted into the canal lumen with sterile 3-mL plastic syringes and 27-gauge needles until the dentin tubes were totally filled. Solutions were removed using sterile paper points ten minutes after placement into the lumen. The specimens were then incubated at 37°C for 28 days to maintain humidity. At experimental times of 0, 7, 14, 21, and 28 days, dentin chips were removed from the canals with sequential sterile low-speed round burs with increasing diameters of ISO sizes: 025, 027, 029, 031, and 033, respectively. Each bur removed approximately 0.1 mm of dentin around the canal. The powder dentin samples obtained with each bur were immediately collected in separate test tubes containing 3 mL of freshly prepared BHI. Thereafter, l00 *μ*L from each test tube was cultured on blood agar. Growing colonies were counted and recorded as CFU.

Analysis of variance and covariance with repeated measures was used (ANOVA) to indicate differences between the experimental groups and the positive control. In addition, one-way ANOVA (Tukey's method) was used to indicate differences within each layer.

## 3. Results

**Figure 2:**
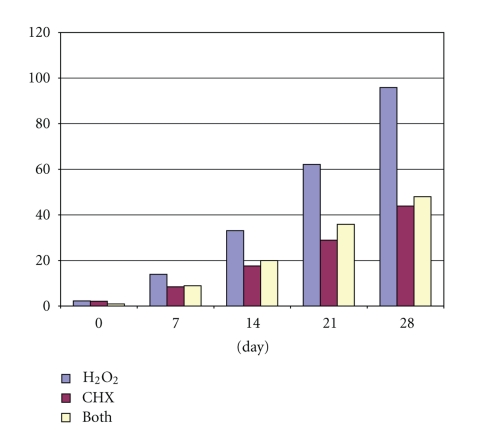
The number of CFU in three experimental groups at five intervals.

The number of CFU obtained from five consecutive dentinal layers was presented in Figure 2. The number of CFU in all three experimental groups was minimum after treatment. The positive control group showed viable bacteria at all experimental times, which indicated the efficiency of the method. In contrast, the negative control group showed no viable bacteria at all experimental times. At the first culture, the CHX + H_2_O_2_ group showed the most effective antibacterial action (*P* < .05). However, at days 7, 14, 21, and 28, the CHX group demonstrated more effective antibacterial action than the other two experimental groups.

## 4. Discussion

*Enterococcus faecalis* is found in 4–40% of primary endodontic infections [20]. However, its frequency in persistent periradicular lesions has been shown to be nine times higher. Its prevalence in root-filled teeth with periradicular lesions using culturing and polymerase chain reaction (PCR) methods is 24–70% and 67–77%, respectively [20]. *E. faecalis* possesses several virulence factors. However, it relies more upon its ability to survive and persist as a pathogen in the root canals of teeth [21]. Furthermore, its capacity to endure prolonged periods of starvation until an adequate nutritional supply becomes available has been demonstrated [21].

Considering the fact that current techniques of root canal instrumentation leave many areas of the root canal completely untouched by the instruments [22], an irrigation solution is required to aid in the debridement of the canals. For improvement of their efficacy, root canal irrigants, the irrigants must be in contact with the dentin walls and debris [23]. It is well known that microorganisms penetrate into dentinal tubules to varying depths [24]. Therefore, if paper point is used to take sample from the root canal system, the possibility of false negative culture is significantly increased. Therefore, in order to decrease the possibility of false negative result of the culturing, it is adviced to cut dentine from root canal walls. There are three ways to achieve this goal: using hand files, using Gates-Glidden drills, and using burs. Hand files and Gates-Glidden drills can be used both *in vitro* and *in vivo*. However, because of the increased risk of perforation, burs should be used *in vitro* only. 

In fact in the present study both the antibacterial substantivity and penetration depth of CHX, H_2_O_2_, and CHX + H_2_O_2_ were evaluated. The use of synergism between any two active agents seems a logical pharmaceutical way to achieve maximal therapeutic effect with minimal side effects. CHX and H_2_O_2_ are both potent antibacterial agents; however, these two agents have considerable side effects; CHX is known to have a bitter taste and to stain teeth [25] while H_2_O_2_ can cause mucosal ulceration [26] and induce pathologic changes that are associated with preneoplastic lesions [27] and pulp cytotoxicity [28]. In order to reduce their side effects, we tested the hypothesis that a combination of subbactericidal concentrations of these two agents may act synergistically. The use of CHX together with H_2_O_2_ has a clinical advantage: it can be postulated that the interactions between HP and CHX may reduce side effects such as teethstaining due to the oxidative properties of HP that may counteract the staining caused by CHX.

Findings showed that the number of CFU of the CHX + H_2_O_2_ was lower than the other two groups, which confirms the synergistic effect between two agents. Heling and Chandler [19] found that at certain concentrations, CHX and H_2_O_2_ had synergistic activity. The method of their study was very similar to the method of present study. Steinberg et al. [18] showed the additive antibacterial effect of CHX and H_2_O_2_ as well. 

The burs used for removing dentine from the lumen of the dentine tubes were selected consecutively and each bur removed a thin layer of 0.1mm thickness. Additionally, the irrigation solutions used (CHX and H_2_O_2_) can kill *E. faecalis* only in direct contact. Therefore, it can be stated that besides antibacterial substantivity, the penetration depth of the irrigants into dentinal tubules was assessed. There is no study on the effect of H_2_O_2_ on the substantivity of CHX. Findings of the present study demonstrated that H_2_O_2_ increased the antibacterial activity of CHX only at the first culture; however, it did not increase its long-term (residual) antibacterial activity.
